# Random transposon mutagenesis identifies genes essential for transformation in *Methanococcus maripaludis*

**DOI:** 10.1007/s00438-023-01994-7

**Published:** 2023-02-24

**Authors:** Dallas R. Fonseca, Madison B. Loppnow, Leslie A. Day, Elisa L. Kelsey, Mohd Farid Abdul Halim, Kyle C. Costa

**Affiliations:** Department of Plant and Microbial Biology, University of Minnesota, Twin Cities, St Paul, MN USA

**Keywords:** Archaea, Natural transformation, Genetics, Competence, Transposon mutagenesis

## Abstract

Natural transformation, the process whereby a cell acquires DNA directly from the environment, is an important driver of evolution in microbial populations, yet the mechanism of DNA uptake is only characterized in bacteria. To expand our understanding of natural transformation in archaea, we undertook a genetic approach to identify a catalog of genes necessary for transformation in *Methanococcus maripaludis*. Using an optimized method to generate random transposon mutants, we screened 6144 mutant strains for defects in natural transformation and identified 25 transformation-associated candidate genes. Among these are genes encoding components of the type IV-like pilus, transcription/translation associated genes, genes encoding putative membrane bound transport proteins, and genes of unknown function. Interestingly, similar genes were identified regardless of whether replicating or integrating plasmids were provided as a substrate for transformation. Using allelic replacement mutagenesis, we confirmed that several genes identified in these screens are essential for transformation. Finally, we identified a homolog of a membrane bound substrate transporter in *Methanoculleus thermophilus* and verified its importance for transformation using allelic replacement mutagenesis, suggesting a conserved mechanism for DNA transfer in multiple archaea. These data represent an initial characterization of the genes important for transformation which will inform efforts to understand gene flow in natural populations. Additionally, knowledge of the genes necessary for natural transformation may assist in identifying signatures of transformation machinery in archaeal genomes and aid the establishment of new model genetic systems for studying archaea.

**Supplementary Information:**

The online version contains supplementary material available at 10.1007/s00438-023-01994-7.

## Introduction

Horizontal gene transfer, the process by which genetic information is transferred independent of inheritance, is a major driver of evolution in microbial populations. This typically occurs via one of the following three mechanisms: conjugation, the uptake of DNA via cell-to-cell contact; transduction, DNA transfer mediated via a virus/phage; or transformation, the direct uptake of DNA from the environment. In the archaea, mechanisms of transduction have been described, conjugation-like systems are known (Greve et al. 2004; Prangishvili 2013; Wolferen et al. 2016; Shalev et al. 2017; Villa et al. 2019), and examples of naturally transformable archaea have been documented (Worrell et al. 1988; Patel et al. 1994; Sato et al. 2003; Lipscomb et al. 2011; Wagner et al. 2017; Fonseca et al. 2020); however, the genetic basis for natural transformation-mediated DNA uptake is not well described. To address this gap, we sought to identify the genes that are required for natural transformation.

Transformation has been well studied in numerous bacteria such as *Vibrio* spp., *Neisseria* spp., and *Bacillus* spp. (Aas et al. 2002; Cehovin et al. 2013; Seitz and Blokesch 2013a; Johnston et al. 2014; Blokesch 2016; Ellison et al. 2018). To enable transformation, a cell enters a physiological state known as the competent state, which can be achieved by sensing cues from other organisms, quorum sensing, starvation, or other environmental stressors (Seitz and Blokesch 2013b). Some organisms (e.g., *Helicobacter pylori*) are hypothesized to be constitutively competent, but external stimuli can alter transformation efficiency in these organisms (Johnston et al. 2014; Corbinais et al. 2017). In the naturally competent archaeon *Methanococcus maripaludis* strain JJ, there was no difference in transformation frequency between exponential phase cell and stationary cells, suggesting that the strain may be constitutively competent (Fonseca et al. 2020). Interestingly, *M. maripaludis* strain S2 is not natively competent in our laboratory conditions; however, it can be induced into the competent state by expression of pilin proteins from a heterologous expression vector (Fonseca et al. 2020), suggesting that there may be regulatory control of competence in *M. maripaludis*.

Once a cell has entered a competent state, transformation begins with the localization of free DNA to the cell surface (in the case of gram-negative bacteria, free DNA is transferred across the outer membrane into the periplasm). DNA localization is facilitated by extracellular appendages such as type IV pili (Aas et al. 2002; Cehovin et al. 2013; Seitz and Blokesch 2013a; Ellison et al. 2018; Piepenbrink 2019), the Flp pilus (Angelov et al. 2015), or the competence pilus (Blokesch 2016). In *M*. *maripaludis* and *Methanoculleus thermophilus*, a type IV-like pilus is essential for natural transformation (Fonseca et al. 2020). We hypothesize that archaeal type IV-like pili and bacterial pili play similar roles in transformation.

After binding by pili, DNA enters the cell. In bacteria, double stranded DNA (dsDNA) is converted to single stranded DNA (ssDNA) before transport by a DNA transporter such as ComEC (Johnston et al. 2014; Pimentel and Zhang 2018). The DNA transporter in naturally competent archaea is unknown, and homologs of ComEC have not been identified in these organisms (Gophna and Altman-Price 2022). Additionally, it is unclear whether dsDNA or ssDNA is the substrate for transformation in archaea. The final step of transformation is the incorporation of DNA into the genome by ssDNA binding proteins and recombinases such as RecA (Claverys et al. 2009; Kidane et al. 2012).

In order to better understand the process of transformation in naturally competent archaea, we sought to determine which genes are essential for transformation. By employing a method for reproducible and high-efficiency transposon mutagenesis in *M. maripaludis*, we developed a screen for identifying defects in natural transformation. From 6144 transformants screened, we identified 71 with insertions corresponding to 25 genes that resulted in a defect in transformation, representing ~ 1.5% of the 1,815 genes encoded within the *M. maripaludis* strain JJ genome (Poehlein et al. 2018). Markerless gene deletion was used to confirm the transformation defect phenotype of these genes. Notably, we found an essential role for a membrane bound TctA-like protein for transformation in two distinct organisms. The TctA-like protein belongs to a family of transporters, but its function has not been characterized in archaea.

## Results

### Optimizing *M. maripaludis* mini-mariner transposon mutagenesis

**Fig. 1:**
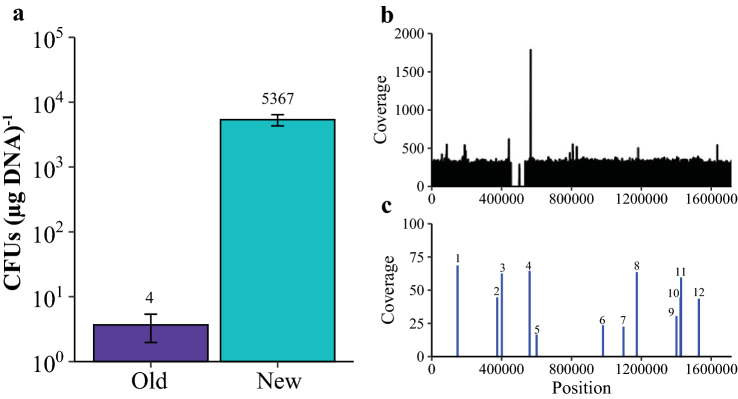
Optimized transposon mutagenesis produces random insertions across genome that can be mapped with whole genome sequencing: **A** Comparison of methods described by Sattler et al*.* (Sattler et al. 2013) and Quitzke et al*.* (Quitzke et al. 2018) [old] and the method described in this study [new]. Data are averages from three independent experiments, and error bars represent one standard deviation around the mean. **B** Coverage distribution of reads mapped from a representative sequencing run. Coverage data created by Bowtie2 alignment of all forward reads to reference genome of *M. maripaludis* JJ (CP026606). **C** Sites of random transposon insertion across genome of 12 randomly picked puromycin grown (containing transposon) colonies. Two Illumina reactions containing 6 random mutants each were sequenced and mapped to identify mini-mariner transposition. Insertion sites position and coverage value were determined through visualization in Geneious prime® (v.2021.0.3). Site determined by presence of AT (known integration site for mariner-based transposition) and where reads of either end of the transposon overlapped

To identify genes important for natural transformation in *M. maripaludis*, we employed random transposon mutagenesis to screen for mutants with a transformation defect. In our hands, previous methods for generating transposon mutants were lower efficiency than other labs have achieved (~ 5 transformants µg DNA^−1^ in our hands vs. 10–30 transformants µg DNA^−1^ reported for mariner transposons (Sattler et al. 2013)), labor intensive, or irreproducible (Sarmiento et al. 2013; Sattler et al. 2013; Quitzke et al. 2018). Thus, we sought to optimize the methods of Sattler et al*.* and Quitzke et al*.* (Sattler et al. 2013; Quitzke et al. 2018) to improve efficiency. Using *HimarI* transposase and a mini-mariner transposon (Lampe et al. 1999), we incubated transposon DNA with at least a twofold stoichiometric excess of purified transposase before transfer into *M. maripaludis* via the polyethylene glycol (PEG) method of transformation (Tumbula et al. 1994). Notably, the protocol we employed differed from the established method in that excess transposase was included in the reaction mixture, incubation time/temperature was optimized before transformation, and a heating step/DNA precipitation that likely inactivated the transposase was omitted. Following the optimized protocol, we observed a transformation efficiency averaging 5,367 colony forming units (CFUs) (µg DNA)^−1^ (Fig. 1A), over 1000-fold more efficient than we could achieve following published methods.

To determine whether our modified method continued to produce random and unique insertions across the genome, and to validate that pooled sequencing would be sufficient to identify all mutants in the pool, we utilized a strategy similar to the previous report that sequenced 10 isolated colonies and mapped 10 unique insertion sites (Sattler et al. 2013). Here, we pooled random colonies and mapped the insertions of 12 mutants across two sequencing reactions (i.e., six transposon insertions were mapped per sequencing run). Each sequencing reaction had relatively uniform coverage across the genome (Fig. 1B). Additionally, mapping reads that aligned to both the genome and to the transposon identified a total of 12 independent insertion sites (Fig. 1C), validating our improved method for generating random transposon insertion mutants and the pooled sequencing approach.

Interestingly, while mapping via whole genome sequencing, we found that our strain of *M. maripaludis* strain JJ (KC13) differed from the published genome (Poehlein et al. 2018) in that it has an apparent ~ 80 kbp deletion (Fig. 1B). Large genome rearrangements are often found between two laboratories working with the same organisms (Periwal and Scaria 2015; Guérillot et al. 2019; Liao et al. 2022), but to ensure that the ~ 80 kbp deletion was not important for the transformation phenotype we observe, we compared the natural transformation efficiency of KC13 to J901, a variant of the sequenced strain (Seyhan et al. 2015). After transformation and selection, we observed that both strains were capable of DNA uptake with a similar efficiency (Figure S1), therefore the deletion present in strain KC13 was not considered further.

### Developing a screen for transformation defects with replicating plasmids

Using transposon mutagenesis, we developed a screen to identify genes important to transformation in *M. maripaludis*. 2496 individual transposon mutants were picked into 96 well plates and grown to stationary phase in the absence of selection. After growth, wells were supplemented with 1 µg mL^−1^ of the replicating plasmid pLW40neo (Dodsworth and Leigh 2006) and incubated for an additional day to allow for DNA uptake and expression of the antibiotic resistant cassette (Fonseca et al. 2020). Cultures were transferred in duplicate into medium containing neomycin and allowed to grow for 2 days, consistent with the time needed for selection of *M. maripaludis* transformants in liquid medium (Fonseca et al. 2020). Any mutants that failed to grow in both wells containing neomycin were considered candidates for a transformation defect. For further validation, candidate mutants were grown in 5 mL liquid culture and rescreened for a transformation defect (Fonseca et al. 2020). Cultures with an OD_600_ > 0.2 at 48 h were considered false positives and were not analyzed further. In total, 46 mutants were selected for further analysis.

**Fig. 2:**
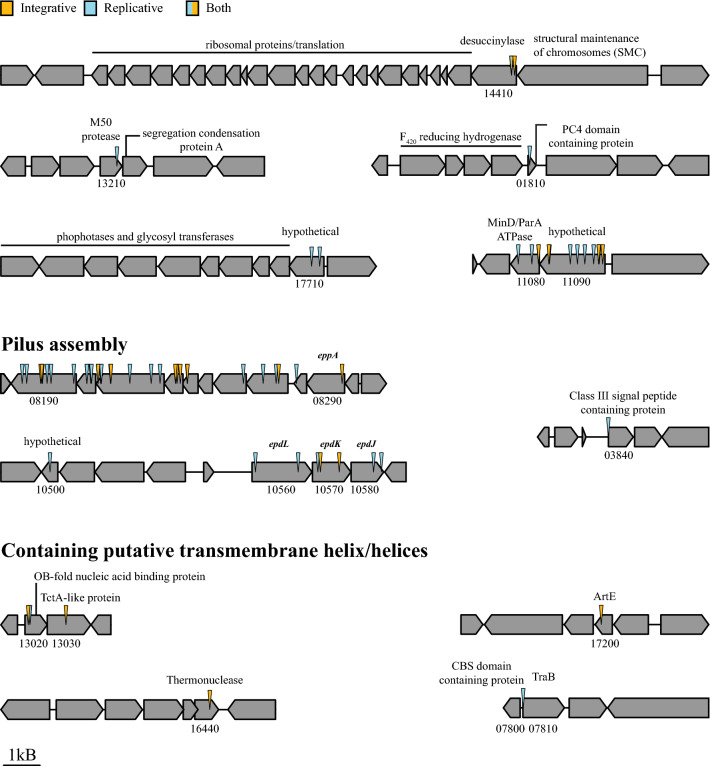
Schematic representation of insertions and respective operons: For each candidate mutant, insertion site was determined via visualization in Geneious prime®(v.2021.0.3). Site determined by presence of AT (known integration site for mariner-based transposition) and where reads of either end of the transposon overlapped. Genes around the insertion are included for context. Genes of interest are referenced using the locus tag format of Poehlein et al. (MMJJ_XXXXX) (Poehlein et al. 2018). Select annotations were added manually based on either NCBI or UniProt annotations. Colors used to mark which DNA substrate used in screen where replicative refers to pLW40neo and integrative refers to pCRUptNeoΔ*flaK*. One insertion in MMJJ_14400 was identified in both substrates, therefore it was colored with both. A table summary for each of these insertions is provided as Table 1

Transposon insertion sites were mapped to the *M. maripaludis* JJ reference genome (Poehlein et al. 2018). Of the 46 mutants sequenced, we identified insertions that likely impacted 21 genes (Fig. 2, Table 1). The majority of mutants had transposon insertions in genes encoding components of the type IV-like pilus, consistent with the importance of pili in natural transformation (Fonseca et al. 2020), and validated that our screen was revealing genes relevant to natural transformation. We additionally identified genes that encoded proteins with at least one predicted transmembrane helix (MMJJ_13020 and MMJJ_07810/7800) and therefore may be performing their function in or at the close vicinity of the membrane. Of the remaining genes identified, some were predicted to be or be in operons with hypothesized DNA/RNA binding proteins, possibly involved in transcription or translation (MMJJ_01810, MMJJ_13210, MMJJ_17710, and MMJJ_14410). Lastly, we identified multiple independent transposon mutant strains with insertions in a two gene operon containing a predicted MinD/ParA ATPase (MMJJ_11080) and a hypothetical protein (MMJJ_11090).

**Table 1:** Insertions identified across both transposon screens

Locus tag	Annotation	Genomic location (nucleotide position) of insertions identified with pLW40neo as substrate	Genomic location (nucleotide position) of insertions identified with pCRuptNeoΔ*flaK* as substrate
MMJJ_01810	PC4 domain-containing protein	172,367	–
MMJJ_03840	Class III signal peptide	365,933	–
MMJJ_07800/ MMJJ_07810	CBS domain-containing protein / Pheromone shutdown-related protein TraB	722,257 (intergenic)	–
MMJJ_08190	*epdI*	754,017; 754,141; 754,574; 754,702; 754,810; 755,436	754,525; 754,557
MMJJ_08200	*epdH*	755,754; 755,796; 755,904; 755,921	–
MMJJ_08210	DUF2341 domain-containing protein	756,173; 756,194; 756,445; 756,977; 757,563; 757,807	756,103; 756,104; 756,456
MMJJ_08230	Uncharacterized protein	758,274	758,229; 758,271; 753,363
MMJJ_08240	*epdC*	–	758,553; 758,560
MMJJ_08260	Uncharacterized protein	760,097	–
MMJJ_08270	*epdF*	760,641; 760,998	761,067
MMJJ_08280	*epdA*	761,556	–
MMJJ_08290	*eppA*	762,641	762,811
MMJJ_10500	Uncharacterized protein	961,509	–
MMJJ_10560	*epdL*	967,156; 968,330	–
MMJJ_10570	*epdK*	968,871	968,941; 969,459
MMJJ_10580	*epdJ*	970,403; 970,619	–
MMJJ_11080	MinD-like ATPase involved in chromosome partitioning or flagellar assembly	1,026,741; 1,027,115; 1,027,298	1,027,299
MMJJ_11090	Uncharacterized protein	1,027,598; 1,028,171; 1,028,366; 1,028,570; 1,028,813; 1,028,953; 1,028,965	1,027,582; 1,028,952; 1,029,061
MMJJ_13020	OB-fold nucleic acid binding domain protein	1,193,088	1,193,041; 1,193,089
MMJJ_13030	Tripartite tricarboxylate transporter TctA family protein	–	1,194,079
MMJJ_13210	Peptidase family M50	1,210,579	–
MMJJ_14410	Putative succinyl-diaminopimelate desuccinylase	1,306,596	1,306,596; 1,306,683
MMJJ_16440	Thermonuclease	–	1,499,668
MMJJ_17200	Archaeosortase family protein ArtE	–	1,571,918
MMJJ_17710	Uncharacterized protein	1,619,404; 1,619,626	–

### Developing a screen for transformation defects with integrating plasmids

**Fig. 3:**
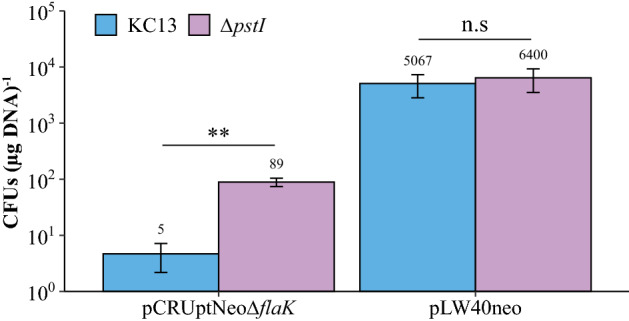
Deletion of MMJJ_06980, a predicted PstI restriction enzyme, increases the transformation efficiency of transformations with integrative plasmids: Transformation efficiencies of KC13 compared to ΔMMJJ_06980 with either pCRUptNeoΔ*flaK* or pLW40neo. Data are averages from three independent experiments, and error bars represent one standard deviation around the mean. Statistics were performed using a two-tailed, equal variance T test. n.s, not significant (*p*. 0.05); * *p* < 0.05; ** *p* < 0.01; *** *p* < 0.001

There is a significant difference between the transformation efficiencies of replicative versus integrative plasmids in *M. maripaludis*. While it was hypothesized that a requirement for recombination of integrating vectors into the genome accounted for this difference (Fonseca et al. 2020), it was recently reported that the presence of PstI restriction sites (5′-CTGCAG-3′) on a plasmid alters transformation efficiency (Bao et al. 2022). The integrating vector pCRUptNeo is typically used for mutagenesis of *M. maripaludis* and contains several PstI restriction sites. Previously, a PstI-like restriction system in *M. maripaludis* JJ (MMJJ_06980) was characterized (Tumbula et al. 1994). Additionally, it was shown that an integrative plasmid (pKAS102) treated with the PstI methylase was partially protected from the native restriction system. Therefore, we hypothesized that deletion of MMJJ_06980 would result in higher natural transformation efficiencies when transforming with pCRUptNeo based integrative vectors. A mutant strain lacking MMJJ_06980 was generated and transformed with the integrating vector pCRUptNeoΔ*flaK* (Fonseca et al. 2020). Transformation of the *M. maripaludis* ∆MMJJ_06980 strain was tenfold more efficient than transformation of the parent strain (Fig. 3) suggesting that the native *M. maripaludis* PstI restriction activity was eliminated in the mutant. There was not a significant difference in transformation efficiency between the parent and the ∆MMJJ_06980 strain when using replicating plasmid pLW40neo (Fig. 3).

The increased transformation efficiency of the ∆MMJJ_06980 strain allowed us to screen transposon mutants for transformation defects with pCRUptNeoΔ*flaK* supplied as transforming DNA to determine if a different set of genes is essential for transformation with integrating vectors. We screened 2880 transposon mutant strains and identified 25 with defects for transformation affecting 14 genes (Fig. 2). Most of the transposon insertions fell into either the same genes or genes in putative operons with those identified in the pLW40neo-based screen (such as MMJJ_13030), suggesting that the transformation pathways are similar regardless of substrate. We additionally obtained transposon insertions in additional membrane associated proteins of unknown function, namely MMJJ_16440 (predicted thermonuclease) and MMJJ_17200 (predicted archaeosortase homolog, *artE*).

### MinD/ParA-like proteins, an uncharacterized candidate archaeaosortase, and a putative membrane bound thermonuclease are required for transformation

To validate both transposon mutagenesis screens, we constructed in-frame deletion mutants for several of the genes identified and tested their ability to take up exogenous DNA. We did not generate in-frame deletion mutants for genes encoding components of the type IV-like pilus as previous work already identified several of these as essential for transformation (Fonseca et al. 2020). While many genes were identified from both screens, all in-frame deletion mutants were tested for transformation defects with pLW40neo as the substrate. Because mutants were defective for natural transformation, complementation plasmids were transferred to cells using a previously established polyethylene glycol-based transformation method (Tumbula et al. 1997).

**Fig. 4:**
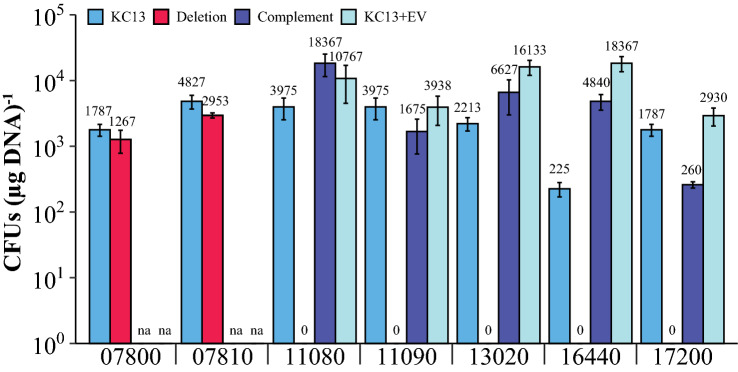
Deletion and complement mutants confirm the role of several candidate genes for transformation: Transformation efficiencies calculated with respect to each of the following gene loci (Genes of interest are referenced using the locus tag format of Poehlein et al. (MMJJ_XXXXX) (Poehlein et al. 2018)). In each, KC13 and deletion were performed using pLW40neo as substrate for transformation, while complement and KC13 + EV (KC13 + pLW40neo [empty vector]) were transformed using pLW40. KC13 and KC13 + EV paired with respective mutants were transformed on the same day with the same transforming DNA. Data are averages from three independent experiments, and error bars represent one standard deviation around the mean. na, not applicable; mutant strains in these genes were not defective for transformation, so complementation of the mutation was not attempted

MMJJ_11080 is a predicted MinD/ParA-like protein. Proteins from this family may function in polar localization of macromolecules. As such, they may be important for DNA segregation to daughter cells or localizing protein complexes to the cell pole (Lutkenhaus 2012). MMJJ_11090, which encodes a hypothetical protein, is in a putative operon with this gene. In-frame deletion mutants of either gene were completely defective for transformation, and *in trans* expression under the control of the high expression P*hmv* promoter (Gardner and Whitman 1999), rescued the transformation defects (Fig. 4).

MMJJ_16440 is predicted to encode a thermonuclease with a secretion signal (Sec/SPII predicted by SignalP 6.0 (Teufel et al. 2022) with 99.7% confidence) at the N-terminus. This specific signal is known to secrete proteins through the Sec pore, then subsequently add a lipid to a cysteine at the cleavage site (in the case of MMJJ_16440, C18) that re-anchors the protein to the outer membrane (Schneewind and Missiakas 2014). MMJJ_17200 is predicted to encode ArtE, an uncharacterized member of the archaeosortase protein family (Haft et al. 2012) where a known member is involved in C-terminal anchoring and processing of archaeal surface proteins (Abdul Halim et al. 2017). In-frame deletion mutants for each of these genes were completely defective for transformation, and complementation of the mutation *in trans* rescued this defect (Fig. 4).

One candidate mutant with a defect in transformation contained a transposon insertion near the 5' end of MMJJ_07810, a gene encoding a putative TraB-like protein. However, a ∆MMJJ_07810 mutant did not have a defect in natural transformation efficiency. We hypothesized that the close proximity of this insertion to the promoter of MMJJ_07800 may have resulted in the observed transformation defect. However, a ∆MMJJ_07800 deletion strain retained wild type transformation efficiency (Fig. 4). It may be that another mutation elsewhere on the genome of the transposon insertion strain resulted in a defect in DNA transfer, but the sequencing analysis was insufficient to identify off target mutations.

### MMJJ_13020 and MMJJ_13030 are essential for transformation and conserved in naturally competent archaea

**Fig. 5:**
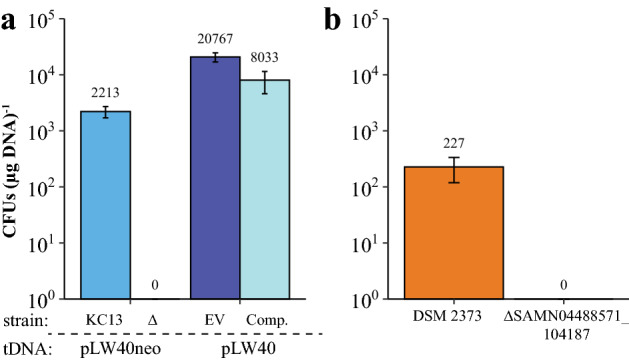
MMJJ_13030 is essential to transformation in *M. maripaludis* and its homolog is essential to transformation in *M. thermophilus* DSM 2373. **A** Transformation efficiencies with and without MMJJ_13030 of *M. maripaludis* JJ. EV denotes KC13 + pLW40neo (empty vector), and Comp. refers to ΔMMJJ_13030 + pLW40neo-MMJJ_13030. KC13 and KC13 + EV paired with respective mutants were transformed on the same day with the same transforming DNA (tDNA). Data are averages from three independent experiments, and error bars represent one standard deviation around the mean. **B** Transformation efficiencies of *M. thermophilus* DSM 2373 and a ∆SAMN04488571_104187 strain transformed on same day with same transforming DNA. Data are averages from three independent experiments, and error bars represent one standard deviation around the mean

Three transposon insertions identified in both screens were in MMJJ_13020 and MMJJ_13030, which are predicted to encode an oligonucleotide/oligosaccharide binding (OB-) fold domain protein with a single transmembrane helix and a TctA-like protein with 11 transmembrane helices, respectively. TctA-like proteins are part of the tripartite tricarboxylate transporter (TTT) protein family (Winnen et al. 2003). Because archaea lack identifiable homologs of the bacterial ComEC family of DNA transporters, we hypothesize that another transporter, possibly a TctA-like protein such as MMJJ_13030, is needed for transformation. *M. maripaludis* strains with in-frame deletions of either MMJJ_13020 (Fig. 4) or MMJJ_13030 (Fig. 5A) were generated and tested for transformation. Both mutant strains were completely defective for transformation, and complementation of the mutation *in trans* rescued this defect.

Several components of the natural transformation machinery are likely conserved in naturally competent archaea. For example, the type IV-like pilus is essential for transformation in both *M. maripaludis and M. thermophilus* (Fonseca et al. 2020). Because TctA-like proteins are found in several members of the Euryarchaeota, and their function in archaea is unknown, we sought to determine if their role in natural transformation is conserved. From a BLASTp analysis using the standard search criterion/databases, we identified a homolog of MMJJ_13030 in *M. thermophilus* (SAMN04488571_104187); a similar BLASTp search did not retrieve a single clear homolog of MMJJ_13020 in this organism.

We generated an *M. thermophilus* mutant with an in-frame deletion of SAMN04488571_104187 and tested the strain for a transformation defect. Because replicating vectors are not available for *M. thermophilus*, we used the integrating plasmid, pJAL1inter, to test for a transformation defect (Fonseca et al. 2020). After incubation with plasmid DNA, we recovered 227 CFUs (µg DNA)^−1^ from wild type *M. thermophilus* on selective media and no transformants from the ΔSAMN04488571_104187 mutant strain (Fig. 5B). Because *M. thermophilus* transformation relies on DNA transfer via natural transformation, and the ΔSAMN04488571_104187 mutant was completely defective for transformation, we were unable to attempt complementation of this mutation. However, these data suggest that the role of the TctA-like proteins may be functionally conserved in naturally competent archaea*.*

## Discussion

The ability to acquire DNA by transformation is a major driver of evolution in microbial populations; however, this process is not well understood in archaea. While type IV-like pili are essential for transformation in both bacteria and archaea (Fonseca et al. 2020), the remaining proteins required for bacterial transformation are not present in archaea. Here, we employed random mutagenesis to identify genes essential to transformation in *M. maripaludis*. While further work is necessary for validating the roles of these genes, we hypothesize that several play a role in transfer of DNA across the cell membrane. For example, it is unclear whether the archaeal DNA transporter requires ssDNA or dsDNA as a substrate; if ssDNA is required, a secreted thermonuclease like MMJJ_16440 may be important for DNA processing.

MMJJ_13030 is annotated as a TctA-like protein, which is a member of the TTT family of organic acid transporters. This family of proteins is widespread across the Euryarchaeota (Winnen et al. 2003), including other competent archaea such as *Thermococcus kodakarensis*, *Methanothermobacter marburgensis*, *Pyrococcus furiosus*, and *Methanococcus voltae* (Fukui et al. 2005; Liesegang et al. 2010; Bridger et al. 2012; Whitman et al. 2015). In the bacterium *Salmonella typhimurium*, TctABC functions as a citrate transporter (Sweet et al. 1979, 1984; Ashton et al. 1980; Somers et al. 1981; Widenhorn et al. 1988); however, archaea lack clear homologs of TctB and TctC (Winnen et al. 2003). Furthermore, archaeal TctA-like proteins typically encode up to 11 transmembrane helices, in contrast to bacterial proteins that encode up to 12 transmembrane proteins. For these reasons, it is likely that archaeal TctA-like proteins carry out a different function from bacterial TctA. In our screen, MMJJ_13030 was the only membrane transporter identified as important for natural transformation and, along with MMJJ_13020, is predicted to bind DNA as determined by analysis using the DRNApred server (Yan and Kurgan 2017). A homolog of MMJJ_13030 in *M. thermophilus*, SAMN04488571_104187, is also essential for transformation, suggesting a conserved role for this protein in transformation across multiple naturally competent archaea. While more work needs to be done to determine the exact role in transformation, these features suggest that MMJJ_13030 may encode the DNA transporter that is specifically required for natural transformation.

We previously showed that type IV-like pili are essential for transformation in both *M. maripaludis* and *M. thermophilus*. Predictably, multiple genes important for expression, processing, and assembly of the type IV-like filament were identified as essential for transformation. Other than pili, a histone protein (TK1413) was shown to be essential for transformation in *T. kodakarensis* (Čuboňová et al. 2012). The authors additionally showed that the deletion of TK1413 resulted in transcriptional changes of various genes in the cell (as determined via microarray analysis). We did not identify genes encoding histones in our screen. To verify that this is not due to a growth defect or failure of the transposon to insert into these relatively small genes, we generated deletion mutants in each of the histone genes (MMJJ_06510 and MMJJ_14920) in *M. maripaludis* and tested their transformation efficiency. We found no observable difference in transformation efficiency of either histone mutant versus KC13 (Figure S3). The essentiality of TK1413 to transformation in *T. kodakarensis* may be due to global transcriptional changes or an indirect effect on the uptake/incorporation of transforming DNA.

Screens for both replicating and integrating vectors identified similar genes important for transformation. pLW40neo is a derivative of pURB500 (Tumbula et al. 1997; Dodsworth and Leigh 2006), which is thought to encode the necessary machinery for DNA partitioning. With integrative plasmids like pCRUptNeo, recombination into the host chromosome is essential for the retention of selective markers; therefore, we initially hypothesized that additional genes would be essential for this step. In bacterial transformation, recombination can be catalyzed by proteins such as RecA (Claverys et al. 2009; Kidane et al. 2012). In *M. maripaludis*, there are multiple predicted recombination machineries present, namely RadA (archaeal homolog of RecA/Rad51), and the Mre11-Rad50 double strand break repair system (Hendrickson et al. 2004). We did not identify genes encoding either of these systems in our screen, possibly due to overlapping functions of recombination systems present in *M. maripaludis* or an essential role for recombination in cell division.

While mapping transposon insertions by whole genome sequencing, we found that strain KC13 lacked ~ 80 kb near the gene encoding tRNA^Ser^ (between nucleotides 452,644 and 534,795) (Figure S2 and Table S1) compared to the reference JJ genome (CP026606 (Poehlein et al. 2018)). In the reference genome, this region is flanked by 56 bp of DNA with 100% sequence identity; therefore, it is likely that homologous recombination between these regions resulted in the loss of DNA. Within this 80 kbp deletion, several of the genes are annotated as encoding uncharacterized proteins or proteins with domains of unknown function. Interestingly, this region also contains predicted integrases, a putative restriction endonuclease and associated DNA methyltransferase, and a type II toxin/anti-toxin system (Table S1). We hypothesize that this region may encode a virus or other mobile genetic element. In any case, KC13 is naturally competent, and the presence of this 80 kb region in another strain of *M. maripaludis* JJ did not impact transformation efficiencies (Figure S1). Therefore, a role for this genomic region was not investigated further as it is not relevant to this study.

Despite the fact that several naturally competent archaea have been characterized (Worrell et al. 1988; Patel et al. 1994; Sato et al. 2003; Lipscomb et al. 2011; Wagner et al. 2017; Fonseca et al. 2020), the genes and proteins essential for transformation have not been characterized. Leveraging transposon mutagenesis and protocols for chemical transformation, we have identified several candidate components of the archaeal natural transformation machinery, including pili, DNA binding proteins, nucleases, and predicted transporters. Knowledge of the genes necessary for transformation will inform future studies directed at biochemically characterizing the transformation machinery, will aid in the identification of competent organisms based on gene content, and broadens understanding of horizontal gene transfer in natural populations.

## Materials and methods

### Strains, media, and growth conditions

Strains used in this study are listed in Table S2. *M. maripaludis* strain JJ was acquired from William Whitman and *M. thermophilus* DSM 2373 was purchased from DSMZ (Leibniz Institut, Deutsche Sammlung von Mikroorganismen und Zellkulturen, Braunschweig, Germany).

*M. maripaludis* strain JJ and its mutants were grown on McCas medium at 37 °C with agitation while *M. thermophilus* DSM 2373 and its mutants were grown in McTry at 55 °C as previously described (Fonseca et al. 2020). When necessary, the following antibiotics were added to media at the noted concentrations: neomycin (1 mg mL^−1^ for liquid medium and 0.5 mg mL^−1^ for plates), puromycin (2.5 μg mL^−1^), or 6-azauracil (0.2 mg mL^−1^). *Escherichia coli* DH5α and Rosetta (pLysS) were grown in lysogeny broth or on lysogeny broth 1.5% agar plates supplemented with ampicillin (50 µg mL^−1^) and incubated at 37 °C.

### Plasmid construction

Primers are listed in Table S3. All mutants/plasmids were made using the methods described in (Fonseca et al. 2020). Briefly, for in-frame genomic deletion mutants in both *M. maripaludis* and *M. thermophilus* 500 bp PCR products of the genomic regions flanking the gene of interest were amplified using primers that encoded 20 bp on either end homologous to pCRUptNeo (Costa et al. 2010) around the XbaI and NotI restriction sites. Additionally, fragments were constructed to retain the first nine and last twelve nucleotides of the open reading frame. Products were assembled with XbaI/NotI digested pCRUptNeo using NEB builder (# E2621) for Gibson assembly (Gibson et al. 2009). pCRUptNeo has features for propagation in *E. coli* (origin of replication and ampicillin resistance gene) and for selection (neomycin resistance) and counterselection (uracil phosphoribosyltransferase) in methanogens. For expression on pLW40neo, genes were PCR amplified with primers that add 20 bp homologous to the regions surrounding AscI and NsiI sites of pLW40neo. These sites place the gene of interest under the control of the *Methanococcus voltae* histone promoter. Assembled constructs were electroporated into *E. coli* DH5α. Transformants were selected on lysogeny broth agar medium containing ampicillin before plasmids were extracted and transferred to methanogens by chemical transformation. All constructs were sequence verified by Sanger sequencing at the University of Minnesota Genomics Center or by long Oxford Nanopore through Plasmidsaurus sequencing service (www.plasmidsaurus.com).

### Purification of HimarI transposase

A modified protocol for purification of *HimarI* transposase from inclusion bodies (Lampe et al. 1996; Sattler et al. 2013) was performed. Plasmid pT7tnp (Sattler et al. 2013) was transferred into *E. coli* Rosetta (pLysS) via electroporation. Cells were allowed to outgrow for ~ 30 min, then 10 µL was inoculated in 5 mL LB + amp and grown overnight. Five µL was subinoculated into four 250 mL LB with ampicillin added flasks and grown with agitation at 37 °C until OD_600_ (Thermo Scientific Genesys 30 spec #840–277,000) was between 0.9–1 before expression was induced with 100 μL of 1.25 M IPTG (0.5 mM final concentration) for 3 h. Cultures were pooled and pelleted by centrifugation at 4000 × g for 10 min. Pellets were suspended in 10 mL of resuspension buffer (20 mM Tris–HCl (pH 7.6), 25% sucrose, 2 mM MgCl_2_, 1 mM dithiothreitol (DTT)), distributed into 1 mL aliquots, and flash frozen in liquid nitrogen for long-term storage.

For purification, 2 mL of cell material was thawed at room temperature (RT) then supplemented with 50 μL of 5 mg mL^−1^ lysozyme and incubated at RT for 5 min. An equal volume of detergent buffer (2 mM Tris–HCl (pH 7.6), 4 mM EDTA, 200 mM NaCl, 1% Triton X-100, 1% deoxycholate) was added and incubated for 20 min at RT. Dnase I (75 units) and Dnase buffer (final 1x) were added and mixed until the solution was no longer viscous, then incubated for an additional 20 min at RT. Pellets containing inclusion bodies were obtained via centrifugation at 16,000 × g for 2 min at RT and all remaining steps were performed at 4 °C.

Pellets were resuspended with 1 mL wash buffer (0.5% Triton X-100, 1 mM EDTA), and pelleted at 16,000 × g for 2 min. Pellets were washed an additional 2 times in 1 mL wash buffer, then 2 more times with 1 mL 6 M Urea. Pellets were resuspended in 500 μl column buffer (4 mM Guanidine HCl, 20 mM Tris HCl (pH 7.6), 50 mM NaCl, 5 mM DTT) and incubated while DEAE-Sepharose Fast Flow (17-0709-10) columns were prepared in column buffer (following manufacturer’s protocol) in disposable columns (Fisher 29,924) with a 2–3 mL bed volume. Pellet resuspensions were briefly centrifuged to remove precipitate, then loaded into equilibrated columns. Fifteen 500 µl fractions were collected and visualized via Coomassie staining of an SDS-PAGE gel. Fractions containing transposase were pooled.

Pooled fractions were dialyzed in 10,000 MWCO slide-a-lyzer dialysis cassettes (Thermo scientific #66,380) in 500 mL dialysis buffer I (10% glycerol, 25 mM Tris HCl (pH 7.6), 50 mM NaCl, 2 mM DTT, 5 mM MgCl_2_) for 5 h with gentle agitation on an orbital rotating shaker. The cassette was then transferred to 500 mL of dialysis buffer II (10% glycerol, 25 mM Tris HCl (pH 7.6), 50 mM NaCl, 0.5 mM DTT, 5 mM MgCl_2_) and gently agitated for an additional 12 h. Sample was removed then centrifuged at 16,000 × g for 2 min to remove precipitate. Supernatant was collected, visualized on an SDS-PAGE gel by Coomassie stain, then the concentration (~ 160 µg mL^−1^) was determined via Bradford assay (Bradford 1976) using Coomassie blue reagent (Thermo Scientific #23,200). Aliquots (25 µl) were flash frozen in liquid nitrogen and stored at -80 °C.

### Optimized transposon mutagenesis

Transposon mutagenesis optimization was based on the methods of Sattler et al. (2013) and Quitzke et al. (2018). The following steps were optimized from the original methods: increased concentration of *HimarI* transposase, elimination of the heat inactivation and ethanol precipitation steps, and a shortened length of incubation. The details of the method are as follows:

On the day of transposon mutagenesis, *M. maripaludis* were subinoculated and grown until an OD_600_ ~ 0.7–0.9. While cultures were growing, the transposon reaction was set up. In a total volume of 50 µl, 2 × transposon reaction buffer (final concentration of reaction: 25 mM HEPES, 100 mM NaCl, 10 mM MgCl_2_, 2 mM dithiothreitol, pH 7.9), 12.5 µg pJJ605, and 10 µg acylated bovine serum albumin (Promega #R396A) were mixed. Reaction was then initiated with 0.2 to 0.8 µg of *HimarI* transposase and incubated at 28 °C for 2 h. Transposon reactions were transferred into *M. maripaludis* using the PEG transformation method (Tumbula et al. 1994) with a 4 h outgrowth. Transformants were selected on McCas + puromycin agar medium and allowed to grow at 37 °C for 4 days in anaerobic incubation vessels.

### 96-well natural transformation screen

Following transposon mutagenesis, individual colonies were picked into single wells of 96 well plates (Fisher #12565501) containing 250 µl of non-selective McCas medium. Plates were allowed to incubate at 37 °C in anaerobic incubation vessels with 20 mL of 25% Na_2_S for 2 days to ensure wells were grown to stationary phase. Each well was supplemented with 50 µL of McCas mixed with pLW40neo (final concentration 1 µg mL^−1^) and the plates were put back in the anaerobic incubation vessels with 15 mL of fresh 25% Na_2_S and allowed to incubate at 37 °C overnight. These plates are referred to as the master plates. 12.5 µl was transferred to one of two replica 96-well plates containing 250 µl McCas + neomycin. Replica plates were performed to remove any false positives that may have arisen from the transfer process. Replica neomycin plates were packaged in anaerobic incubation vessels supplemented with 20 mLs of 25% Na_2_S and allowed to grow at 37 °C for 2 days for pLW40neo or 3 days with pCRUptNeoΔ*flaK* while master plates were packaged into anaerobic incubation vessels without any Na_2_S and pressurized to 140 kpa with N_2_:CO_2_ (80:20) at room temperature. Anaerobic incubation vessels were opened and screened for presence or absence of turbidity by eye. Wells where both plates had either no or unclear growth were marked as candidate competence defective mutants.

Cultures from the master plate that corresponded to the candidate mutants were transferred to 5 mL McCas medium and grown for 24 h. Any tubes that were not fully grown in 24 h were marked as slow growth mutants and were excluded from further characterization. Remaining candidates were rescreened for a natural transformation defect (Fonseca et al. 2020) with 4 h of outgrowth. 0.2 mL of outgrowth were inoculated into 5 mL McCas + neomycin and allowed to grow shaking at 37 °C. Optical density at 600 nm (OD_600_) was measured at 48 h. A wild-type culture of *M. maripaludis* strain JJ transformed with 5 µg of pLW40neo typically grows to stationary (OD_600_ ~ 1.0) in ~ 40 h with a transformation efficiency average of ~ 2000 CFUs (µg DNA)^−1^, so 48 h was used to try and narrow candidates to only include those essential to transformation. Candidate mutants with an OD_600_ > 0.2 (limit of detection for growth) at 48 h were considered false positives, while the rest were prepared for sequencing described below.

### Genomic DNA extractions, sequencing, and mapping

One mL of culture was collected from each mutant and genomic DNA (gDNA) was extracted using Qiagen Blood and Tissue Dneasy kit (#69,504). Up to 6 cultures were pooled during the lysis step. Purified gDNA pools were submitted to the Microbial Genome Sequencing center (MiGS) for 2 × 151 bp sequencing using the NextSeq 2000 platform.

For all sequencing alignments, forward sequencing reads were analyzed by local alignment using Bowtie2 (v.2.3.4.1) with default parameters (Langmead and Salzberg 2012). The resulting alignments were visualized using Geneious prime® (v.2021.0.3). For identification of transposon insertion loci, reads were first mapped to the plasmid pJJ605 sequence, which contains the transposon. Reads that aligned to pJJ605 were then mapped to the *M. maripaludis* strain JJ genome (Accession CP026606). With this approach, non-transposon reads that corresponded to the P*mcrB* and in the case of integrative plasmids, reads that are shared between pCRUptNeoΔ*flaK*, and pJJ605 were observed. These reads did not share the same insertion pattern as transposon reads, therefore were excluded from analysis. In all samples analyzed, the number of mutants that were pooled equaled the number insertions mapped, validating the sequencing approach.

### Transformation of *M. maripaludis* and *M. thermophilus*

Chemical transformations of *M. maripaludis* were performed as previously described in (Tumbula et al. 1994) with the variations described in (Fonseca et al. 2020). All natural transformations for both *M. maripaludis* and *M. thermophilus* were performed as described (Fonseca et al. 2020).

## Supplementary Information

Below is the link to the electronic supplementary material.

Supplementary file1 (DOCX 144 KB)
