# Supplementary material for: Random transposon mutagenesis identifies genes essential for transformation in Methanococcus maripaludis
Source: Mol Genet Genomics. 2023 Feb 24;298(3):537–48. doi: 10.1007/s00438-023-01994-7 (PMC10133366; doi:10.1007/s00438-023-01994-7)
Supplement: Supplementary file 1 — Supplementary file1 (DOCX 144 KB) [file 438_2023_1994_MOESM1_ESM.docx]

**Supplementary Materials**


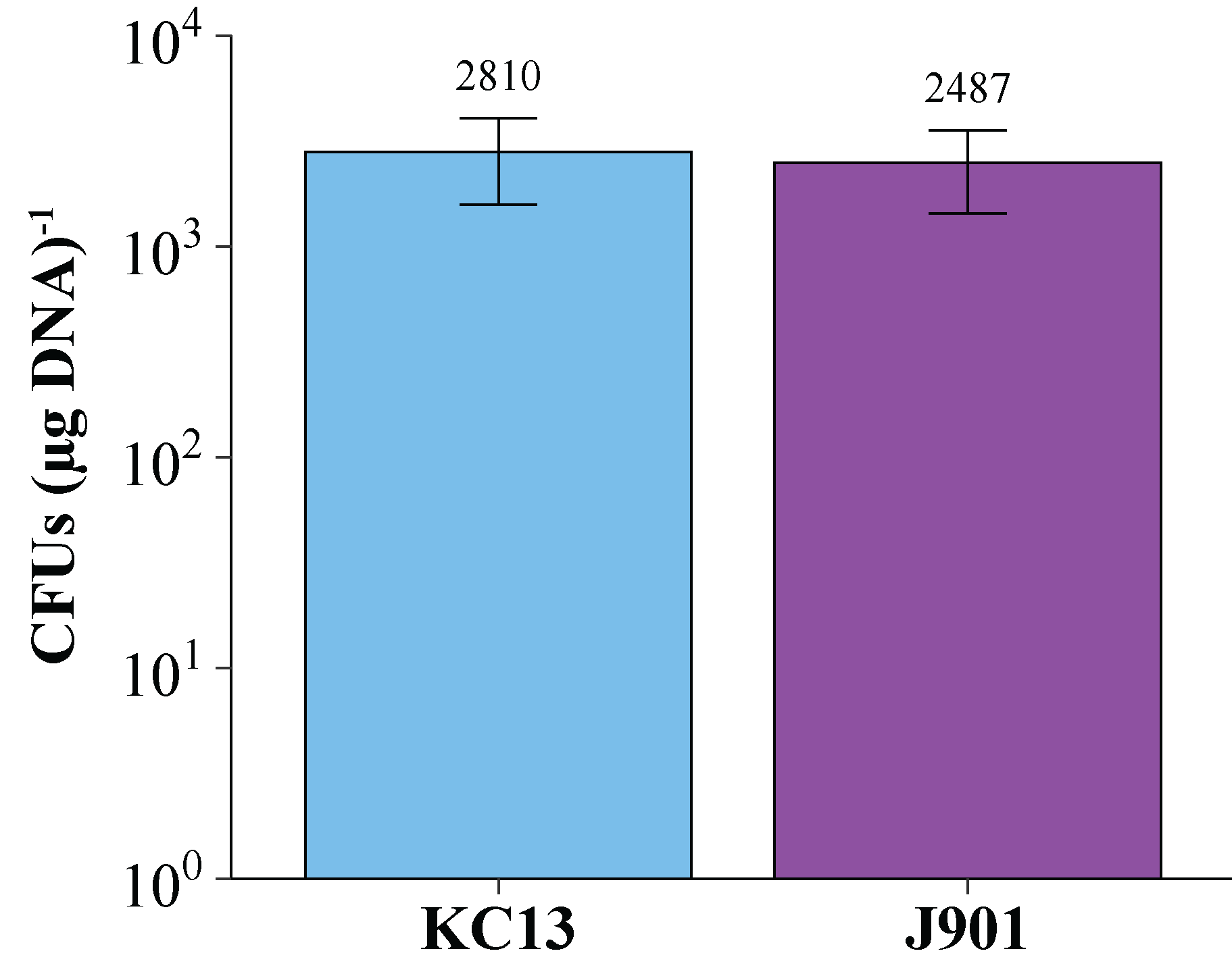


**Figure S1 – Transformation Efficiency of KC13 vs J901:** Transformation efficiencies of KC13 compared to J901 transformed with pLW40neo. Data are averages from three independent experiments, and error bars represent one standard deviation around the mean. A two-tailed, equal variance T test was performed with p=0.792.


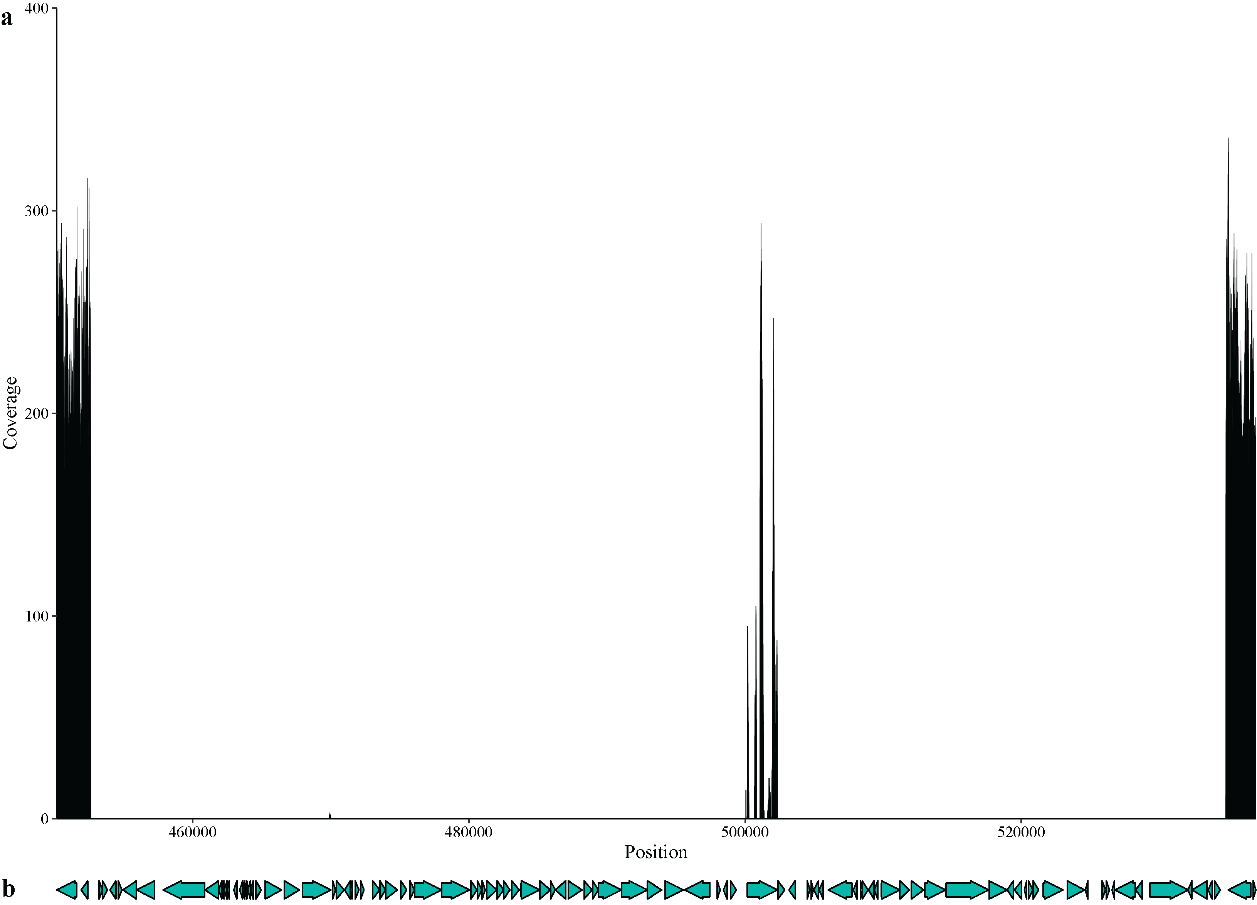


**Figure S2 – Genome coverage and gene content around KC13 deletion:** a) Representative coverage data of a single whole genome sequencing run from bp 450159 to 536989 (coverage to the rest of the genome presented in figure 1b). b) gene schematic of the genes within the coverage region. A table with annotations for each of the genes in the region lacking coverage is provided as table S1.

**
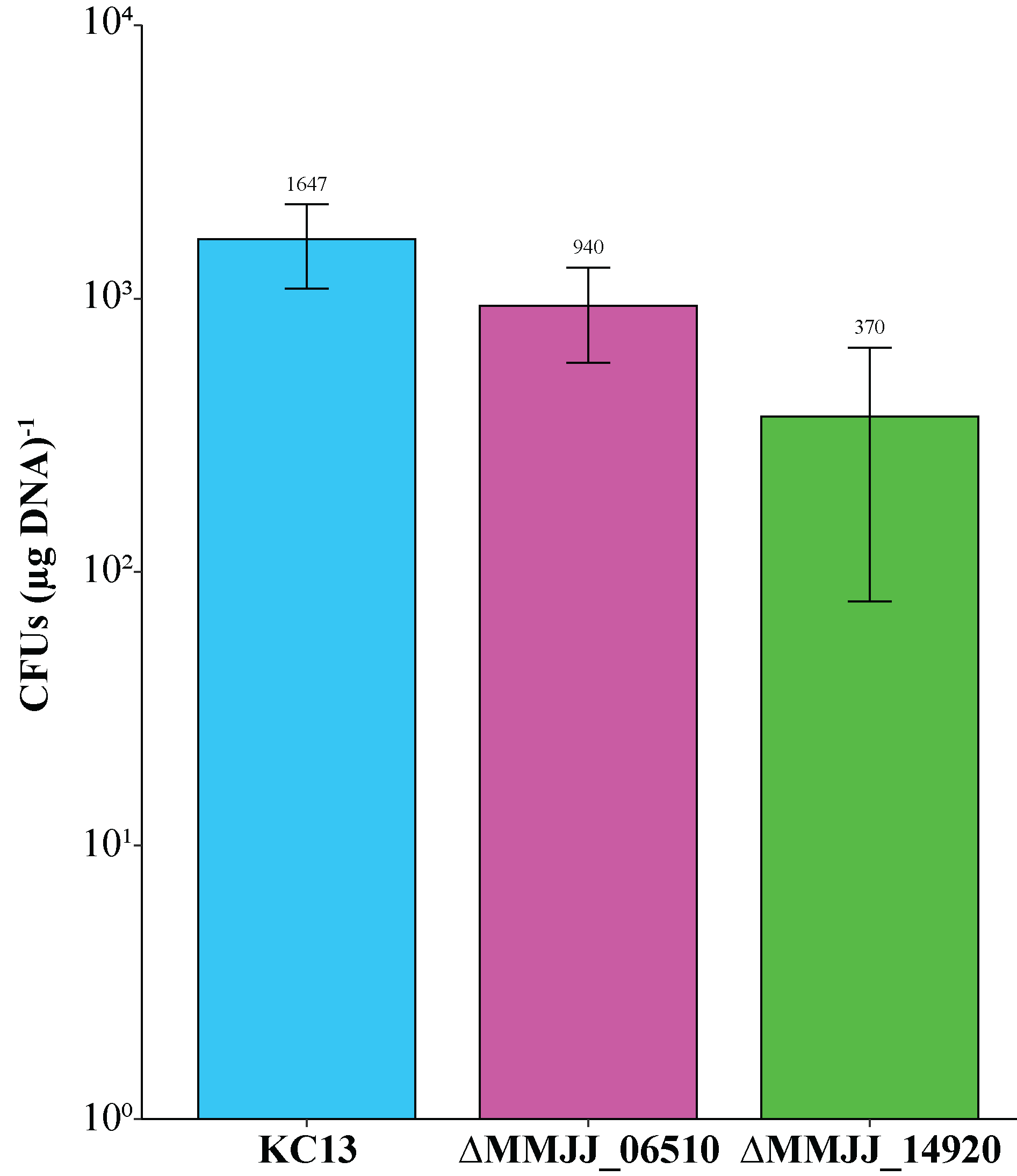
**

**Figure S3 – Transformation efficiency of KC13 compared to histone gene deletion mutants:**

Transformation efficiencies of KC13, and deletion mutants lacking genes for either histone (MMJJ_06510 or MMJJ_14920). pLW40neo was used as substrate for transformation. Data are averages from three independent experiments, and error bars represent one standard deviation around the mean. An ANOVA was performed with a resulting p=0.06.

**Table S1 – Gene context of *M. maripaludis* JJ (CP026606) lacking coverage in genome sequencing (from 452644 – 534795)**: Locus tags of genes present within the region lacking coverage in the genome sequencing. Annotations provided were curated from Uniprot.org. The locus highlighted in red represents the region with incomplete coverage within the ~80 kbp deletion.

| **Locus Tag** | **Annotation from Uniprot** |
| --- | --- |
| MMJJ_04800 | Transcriptional regulator |
| MMJJ_04810 | Type II toxin-antitoxin system RelE/ParE family toxin |
| MMJJ_04820 | GreA_GreB domain-containing protein |
| MMJJ_04830 | Bacterial regulatory protein, arsR family |
| MMJJ_04840 | DUF1616 domain-containing protein |
| MMJJ_04850 | DNA_LIGASE_A3 domain-containing protein |
| MMJJ_04860 | Ankyrin repeat domain-containing protein |
| MMJJ_04870 | Integrase/recombinase XerD, Tyrosine recombinase XerC |
| MMJJ_04880 | Integrase |
| MMJJ_04890 | ACT domain-containing protein |
| MMJJ_04900 | DUF3006 domain-containing protein |
| MMJJ_04910 | Malate dehydrogenase |
| MMJJ_04920 | Type II toxin-antitoxin system RelE/ParE family toxin |
| MMJJ_04930 | Putative transposon-encoded protein |
| MMJJ_04940 | Uncharacterized protein |
| MMJJ_04950 | Zn finger protein HypA/HybF involved in hydrogenase expression |
| MMJJ_04960 | TfoX_N domain-containing protein |
| MMJJ_04970 | DNA-binding HxlR family transcriptional regulator |
| MMJJ_04990 | TIR domain-containing protein |
| MMJJ_05000 | TIR domain-containing protein |
| MMJJ_05010 | S_layer_C domain-containing protein |
| MMJJ_05020 | Superfamily II DNA or RNA helicase, Type III restriction enzyme, res subunit |
| MMJJ_05030 | Uncharacterized protein |
| MMJJ_05040 | Transcription elongation factor GreA |
| MMJJ_05050 | DNA-binding HxlR family transcriptional regulator |
| MMJJ_05060 | DUF3796 domain-containing protein |
| MMJJ_05080 | Borrelia ORF-A superfamily |
| MMJJ_05090 | Uncharacterized protein |
| MMJJ_05100 | DUF4065 domain-containing protein |
| MMJJ_05110 | DUF4365 domain-containing protein |
| MMJJ_05120 | PEGA domain-containing protein |
| MMJJ_05130 | Tetratricopeptide (TPR) repeat protein |
| MMJJ_05140 | Small-conductance mechanosensitive channel |
| MMJJ_05150 | Glucan phosphoethanolaminetransferase (Alkaline phosphatase superfamily) |
| MMJJ_05160 | Putative neutral ceramidase superfamily lipid hydrolase |
| MMJJ_05180 | DUF4352 domain-containing protein |
| MMJJ_05190 | ABC-type xylose transport system permease subunit |
| MMJJ_05200 | DUF4430 domain-containing protein |
| MMJJ_05210 | Cell division protein FtsB |
| MMJJ_05220 | AAA-like domain protein |
| MMJJ_05230 | Uncharacterized protein |
| MMJJ_05240 | Uncharacterized protein |
| MMJJ_05250 | Putative amino-acid-binding protein YxeM |
| MMJJ_05260 | Type II methyltransferase, 2.1.1.113, N-4 cytosine-specific methyltransferase |
| MMJJ_05270 | DNA-invertase hin |
| MMJJ_05280 | DUF1273 family protein |
| MMJJ_05290 | NurA domain protein |
| MMJJ_05300 | AAA-like domain protein |
| MMJJ_05310 | Radical SAM protein |
| MMJJ_05320 | CvpA family protein |
| MMJJ_05330 | Recombination protein F |
| MMJJ_05340 | Uncharacterized protein |
| MMJJ_05350 | Transcriptional regulator PadR-like family protein |
| MMJJ_05360 | GreA_GreB domain-containing protein |
| MMJJ_05370 | Methyl-accepting chemotaxis protein, Methyl-accepting chemotaxis protein McpB |
| MMJJ_05380 | Conserved domain protein |
| MMJJ_05390 | DNA-binding MarR family transcriptional regulator, Transcriptional regulator SlyA |
| MMJJ_05400 | HTH_45 domain-containing protein |
| MMJJ_05410 | WYL domain-containing protein |
| MMJJ_05420 | Guanylate cyclase domain-containing protein |
| MMJJ_05430 | DNA-binding HxlR family transcriptional regulator, MarR family protein |
| MMJJ_05440 | Transcription elongation factor GreA |
| MMJJ_05450 | DUF2178 domain-containing protein |
| MMJJ_05460 | Transcriptional regulator |
| MMJJ_05470 | MAC/Perforin domain protein |
| MMJJ_05480 | Cytolethal distending toxin subunit B |
| MMJJ_05490 | Putative transport protein |
| MMJJ_05500 | Site-specific DNA-methyltransferase (adenine-specific), 2.1.1.72 |
| MMJJ_05510 | Type I site-specific deoxyribonuclease, 3.1.21.3 |
| MMJJ_05520 | EcoKI restriction-modification system protein HsdS |
| MMJJ_05530 | EVE domain-containing protein |
| MMJJ_05540 | Transcription factor |
| MMJJ_05550 | Phage protein |
| MMJJ_05560 | CopG family transcriptional regulator |
| MMJJ_05570 | Transcription elongation factor GreA |
| MMJJ_05580 | 1-phosphatidylinositol phosphodiesterase, 4.6.1.13 |
| MMJJ_05590 | Clostridium P-47 protein |
| MMJJ_05600 | Integral membrane protein |
| MMJJ_05610 | DUF3892 domain-containing protein |
| MMJJ_05620 | Uncharacterized protein |
| MMJJ_05630 | HD domain-containing protein |
| MMJJ_05640 | Transporter |
| MMJJ_05650 | Beta-barrel assembly-enhancing protease, 3.4.-.- |
| MMJJ_05660 | Transcriptional regulator PadR-like family protein |
| MMJJ_05670 | Ras-GEF domain-containing protein |
| MMJJ_05680 | HTH_34 domain-containing protein |
| MMJJ_05690 | Transcription elongation factor GreA |

**Table S2 – Archaeal strains used in this study:**

| **Strain Name** | **Genotype** | **Reference** |
| --- | --- | --- |
| KC13 | *M. maripaludis* strain JJ*Δupt (MMJJ_05990)* with 80 kbp deletion | (Fonseca *et al*. 2020) |
| J901 | *M. maripaludis* strain JJ*Δupt (MMJJ_05990)* | (Seyhan *et al*. 2015) |
| KC78 | KC13Δ*pstI* (MMJJ_06980) | This Study |
| KC120 | KC13ΔMMJJ_07800 | This Study |
| KC87 | KC13ΔMMJJ_07810 | This Study |
| KC103 | KC13ΔMMJJ_11080 | This Study |
| KC104 | KC13ΔMMJJ_11090 | This Study |
| KC123 | KC13ΔMMJJ_13020 | This Study |
| KC122 | KC13ΔMMJJ_13030 | This Study |
| KC89 | KC13ΔMMJJ_16440 | This Study |
| KC121 | KC13ΔMMJJ_17200 | This Study |
| DSM2373 | *M. thermophilus* DSM 2373 WT | (Rivard and Smith 1982) |
| KC138 | DSM2373ΔSAMN04488571_104187 | This Study |
| KC19 | KC13+pLW40neo | This Study |
| KC105 | KC103+pLW40neo-MMJJ_11080 | This Study |
| KC106 | KC104+pLW40neo-MMJJ_11090 | This Study |
| KC74 | KC123+pLW40neo-MMJJ_13020 | This Study |
| KC73 | KC122+pLW40neo-MMJJ_13030 | This Study |
| KC107 | KC89+pLW40neo-MMJJ_16440 | This Study |
| KC130 | KC121+pLW40neo-MMJJ_17200 | This Study |
| KC144 | KC13ΔMMJJ_06510 | This Study |
| KC145 | KC13ΔMMJJ_14920 | This Study |

**Table S3 – Primers used in this study**

| **KC78** | DF1-257 | tccatcacactggcggccgcttgtggttacataattttaatgacg |
| --- | --- | --- |
|  | DF1-258 | ttatcgaggaccctttcccatactacccttccacaaaattagatat |
|  | DF1-259 | atgggaaagggtcctcgataaattaatgctatcttaaaattatttt |
|  | DF1-260 | cgaattgggccctctagaggcacaaaataatccagcgcatctt |
| **KC120** | DF1-283 | tccatcacactggcggccgcaaaaagtcaaatttttctttaagtg |
|  | DF1-284 | ttaaatcattgcaaaaatcattcttccaccagattatttatcaaca |
|  | DF1-285 | atgatttttgcaatgatttaaaggtgaagtttaatgaagttgaatt |
|  | DF1-286 | ggcgaattgggccctctagactaaaatcaattttatcttttcctt |
| **KC87** | DF1-267 | atcacactggcggccgcatcccaaccaatttctcgccttcaacta |
|  | DF1-268 | ttataaaaatcttatatacattttaccaccaatatatctagtagctatatc |
|  | DF1-269 | atgtatataagatttttataatgcgtttgtattttccagaaatcta |
|  | DF1-270 | ggcgaattgggccctctagagaatcgatatttaacgatttatcgt |
| **KC103** | DF1-216 | atcacactggcggccgcttcaataaatacaataagaatatta |
|  | DF1-217 | ttaaaattcgtccaatctcatactaccacttcctagttactaagtg |
|  | DF1-218 | atgagattggacgaattttaaaaaatcaatttattacttttttgg |
|  | DF1-219 | ggcgaattgggccctctagaaggacccaagttttgtttcaaagat |
| **KC104** | DF1-220 | atcacactggcggccgcttcgtcaaattcaagcctgatttc |
|  | DF1-221 | ctagttactaagcagcttcatcaaatcaccgcagtattgttaataa |
|  | DF1-222 | atgaagctgcttagtaactaggaagtggtagtatgagattgggatt |
|  | DF1-223 | ggcgaattgggccctctagatattgcgtaaagaagctcttttgtt |
| **KC123** | DF1-224 | atcacactggcggccgcaatccagtaactaaaccatgggttttt |
|  | DF1-225 | ttaatccctttcagacgacaatttaacccctcattaattatgtcct |
|  | DF1-226 | ttgtcgtctgaaagggattaaatgattaataatttattctttttaa |
|  | DF1-227 | ggcgaattgggccctctagaataattcccaaaattcctgaaaata |
| **KC122** | DF1-228 | atcacactggcggccgctttctgatttaaatgaaggtgatta |
|  | DF1-229 | ttaaatttttaaattaatcatttaatccctttcttcacagtaactg |
|  | DF1-230 | atgattaatttaaaaatttaaaaaagagttattcaataagatagaagt |
|  | DF1-231 | ggcgaattgggccctctagaactcctctttgaaaaattacaatta |
| **KC89** | DF1-277 | atcacactggcggccgcaaaaagggtagttgaaagactttctttaa |
|  | DF1-278 | ttaattgttgtattttttcataataaaccctcaaaaatccagttct |
|  | DF1-279 | atgaaaaaatacaacaattaatattttaaaataggaatcatttttt |
|  | DF1-280 | cgaattgggccctctagattgcaggaggactttgtgagagttct |
| **KC121** | DF1-287 | atcacactggcggccgcttcttgagatgaaaaataaaggaat |
|  | DF1-288 | ttataatctttttttattcatgttttatcatgttgcatgtcatgatatatcc |
|  | DF1-289 | atgaataaaaaaagattataaacacggttgctgatattactaattac |
|  | DF1-290 | ggcgaattgggccctctagagaattttattgaatcttaacttttt |
| **KC138** | ML1-25 | tatatctagaacagacgtactccttgttgtg |
|  | ML1-26 | gtcgatataagagcgacaaaatatcggtcagagcgg |
|  | ML1-27 | ctgaccgatattttgtcgctcttatatcgaccaagcc |
|  | ML1-28 | atatgcggccgcctactccggaagcgagcag |
| **KC105** | DF1-247 | aactaataggtgaaatgcatgagattgggattttacaacatacaa |
|  | DF1-248 | acagatctcctaggcgcgccttaaaattcgtcttcttcaacatcc |
| **KC106** | DF1-249 | aactaataggtgaaatgcatgaagctgaaattttttggcttgatt |
|  | DF1-250 | acagatctcctaggcgcgccctagttactaagtgaaattgtatct |
| **KC74** | ML1-31 | atatgggccctcgtctttaatattaaatgataaaaaaataac |
|  | ML1-32 | atatggcgcgccttaatccctttcttcacag |
| **KC73** | ML1-29 | atatgggcccattaataatttattctttttaatttttgg |
|  | ML1-30 | atatggcgcgccttaaatttttaaaaaataaagaattgttg |
| **KC107** | DF1-281 | aactaataggtgaaatgcatgaaaaaaacatgttttttagcccca |
|  | DF1-282 | acagatctcctaggcgcgccttaattgttgtaattccaaaggccg |
| **KC130** | DF1-295 | aactaataggtgaaatgcatgaataaaaaatttaacgaatatttt |
|  | DF1-296 | acagatctcctaggcgcgccttataatctttttttgattttcgac |

**Table S4 – Plasmid backbones used in this study**

| **Name** | **Description** | **Reference** |
| --- | --- | --- |
| pLW40 | Replicative plasmid in *M. maripaludis*. Contains puromycin resistance cassette. Used as DNA substrate for transformation of complement strains. | (Dodsworth and Leigh 2006) |
| pLW40neo | Replicative plasmid in *M. maripaludis*. Contains neomycin resistance cassette. Used as DNA substrate for transformation of deletion mutants/transposon mutants. Also used as a backbone for complementation constructs (under control of p*hmv* promoter). | (Dodsworth and Leigh 2006) |
| pCRUptNeo | Vector backbone for generating deletion mutants. | (Costa *et al*. 2010) |
| pCRUptNeo*ΔflaK* | DNA substrate for testing transformation of *M. maripaludis* with integrative vectors. Plasmid integrates either upstream or downstream of the flagella peptidase, *flaK*. | (Fonseca *et al*. 2020) |
| pJJ605 | Non-replicating plasmid (in *M. maripaludis*) carrying mini-mariner transposon. | (Sattler *et al*. 2013) |
| pT7Tnp | Plasmid carrying HimarI hyperactive transposase expressed under the inducible λ *P_L_* promoter | (Sattler *et al*. 2013) |
| pJAL1inter | DNA substrate for testing transformation of *M. thermophilus*. Plasmid integrates into an ~500bp upstream of SAMN04488571_11135. | (Fonseca *et al.* 2020) |

**Supplemental References:**

Costa KC, Wong PM, Wang T, *et al* (2010) Protein complexing in a methanogen suggests electron bifurcation and electron delivery from formate to heterodisulfide reductase. Proc. Natl. Acad. Sci. U S A. 107:11050–11055.

Dodsworth JA, Leigh JA (2006) Regulation of nitrogenase by 2-oxoglutarate-reversible, direct binding of a PII-like nitrogen sensor protein to dinitrogenase. Proc. Natl. Acad. Sci. U S A. 103:9779–9784.

Fonseca DR, Halim MFA, Holten MP, Costa KC (2020) Type IV-like pili facilitate transformation in naturally competent archaea. J. Bacteriol. 202:e00355-20.

Rivard CJ, Smith PH (1982) Isolation and characterization of a thermophilic marine methanogenic bacterium, *Methanogenium thermophilicum* sp. nov.†. Int. J. Syst. Evol. Microbiol. 32:430–436.

Sattler C, Wolf S, Fersch J, *et al* (2013) Random mutagenesis identifies factors involved in formate-dependent growth of the methanogenic archaeon *Methanococcus maripaludis*. Mol. Gen. Genomics. 288:413–424.

Seyhan D, Jehmlich N, von Bergen M, *et al* (2015) Selenocysteine-independent suppression of UGA codons in the archaeon *Methanococcus maripaludis*. Biochimica et Biophysica Acta (BBA) - General Subjects 1850:2385–2392.
